# Modulation of the Cardiac Myocyte Action Potential by the Magnesium-Sensitive TRPM6 and TRPM7-like Current

**DOI:** 10.3390/ijms22168744

**Published:** 2021-08-14

**Authors:** Asfree Gwanyanya, Inga Andriulė, Bogdan M. Istrate, Farjana Easmin, Kanigula Mubagwa, Regina Mačianskienė

**Affiliations:** 1 Department of Cardiovascular Sciences, KU Leuven, 3000 Leuven, Belgium; asfree.gwanyanya@uct.ac.za (A.G.); istratem.bogdan@yahoo.com (B.M.I.); feasmin87@gmail.com (F.E.); kanigula.mubagwa@kuleuven.be (K.M.); 2 Department of Human Biology, University of Cape Town, Cape Town 7925, South Africa; 3 Institute of Cardiology, Lithuanian University of Health Sciences, 50103 Kaunas, Lithuania; inga.andriule@lsmuni.lt; 4 Department of Basic Sciences, Faculty of Medicine, Université Catholique de Bukavu, Bukavu, Congo

**Keywords:** TRPM6, TRPM7, ion channel, action potential, cardiac, magnesium, myocyte

## Abstract

The cardiac Mg^2+^-sensitive, TRPM6, and TRPM7-like channels remain undefined, especially with the uncertainty regarding TRPM6 expression in cardiomyocytes. Additionally, their contribution to the cardiac action potential (AP) profile is unclear. Immunofluorescence assays showed the expression of the TRPM6 and TRPM7 proteins in isolated pig atrial and ventricular cardiomyocytes, of which the expression was modulated by incubation in extracellular divalent cation-free conditions. In patch clamp studies of cells dialyzed with solutions containing zero intracellular Mg^2+^ concentration ([Mg^2+^]_i_) to activate the Mg^2+^-sensitive channels, raising extracellular [Mg^2+^] ([Mg^2+^]_o_) from the 0.9-mM baseline to 7.2 mM prolonged the AP duration (APD). In contrast, no such effect was observed in cells dialyzed with physiological [Mg^2+^]_i_. Under voltage clamp, in cells dialyzed with zero [Mg^2+^]_i_, depolarizing ramps induced an outward-rectifying current, which was suppressed by raising [Mg^2+^]_o_ and was absent in cells dialyzed with physiological [Mg^2+^]_i_. In cells dialyzed with physiological [Mg^2+^]_i_, raising [Mg^2+^]_o_ decreased the L-type Ca^2+^ current and the total delayed-rectifier current but had no effect on the APD. These results suggest a co-expression of the TRPM6 and TRPM7 proteins in cardiomyocytes, which are therefore the molecular candidates for the native cardiac Mg^2+^-sensitive channels, and also suggest that the cardiac Mg^2+^-sensitive current shortens the APD, with potential implications in arrhythmogenesis.

## 1. Introduction

The cardiac cation channels involved in electrical activity and ion homeostasis include well-known ion-selective channels, as well as ion nonselective channels. Apart from the Na^+^- and K^+^-permeable pacemaker or funny (I_f_) channels [1], not many nonselective cardiac cation channels have been fully characterized; hence, their molecular identities remain uncertain. During the past two decades, there has been a growing interest in transient receptor potential (TRP) proteins as molecular candidates for native cation nonselective channels, including those found in the heart [2]. TRP channels are a large superfamily of proteins expressed in several tissues where they are involved in diverse signaling processes and in disease [3]. Among the various TRP channels expressed in the cardiovascular system, canonical (e.g., TRPC1 and C3–C7), melastatin (e.g., TRPM4 and M7), vanilloid (e.g., TRPV1 and V2), and polycystin (e.g., TRPP1/2) channels are present in the heart and are implicated in the physiological functions and in cardiac abnormalities such as arrhythmogenesis and heart failure [4,5]. The role of cardiac TRP channels has been explored in fibroblasts and in pacemaker cells (see reference [3]) but much less in cardiomyocytes. The few TRPs that have been electrophysiologically explored in cardiac myocytes include TRPC1 [6], TRPC3/6/7 [7,8], TRPV4 [9], TRPM4 [10], TRPM7 [11,12], and TRPP1/2 [13].

We have previously characterized cardiac Mg^2+^-sensitive channels [14,15] with biophysical properties similar to those of the heterologous TRPM6 and TRPM7 channels, both of which are involved in the homeostasis of Mg^2+^ and other divalent cations [16,17,18,19,20]. Like the heterologously expressed TRPM6 and TRPM7 channels, the cardiomyocyte TRPM6- and TRPM7-like channels are typically activated by low intracellular Mg^2+^ concentration ([Mg^2+^]_i_) conditions and conduct small inward currents carried by divalent cations and large outward currents carried by monovalent cations. These cardiomyocyte Mg^2+^-sensitive channels have been detected in various species, including humans, rats, pigs, guinea pigs, and mice. However, the nature of the proteins underlying the cardiac TRPM6- and TRPM7-like currents and the functional consequences of their cation fluxes have remained unclear.

Whereas TRPM7 protein expression has been systematically detected in cardiac tissues or cells, until recently, information regarding the TRPM6 channel protein expression in the heart has remained scant and has concerned only the right atrium [21]. However, very recent data on human atrial/ventricular cardiomyocytes and tissues has highlighted the co-expression of TRPM6 and TRPM7 in cardiomyocytes from all chamber walls of the human heart [22]. As far as function is concerned, we have previously shown the cardiac Mg^2+^-sensitive channels to be permeable to Ca^2+^ and Mg^2+^ [15] and proposed that, at the resting membrane potential, the inward flow of divalent cations into cells through the channels could have an effect on the intracellular concentrations of the divalent cations (see reference [23]). On the other hand, the contribution of the TRPM6- and TRPM7-like currents to the cardiomyocyte electrical activity is unknown. The possibility that monovalent cation effluxes through Mg^2+^-sensitive channels at positive potentials could contribute to action potentials has not been tested.

Here, we investigated the expression profile of TRPM6 and TRPM7 proteins in pig cardiac myocytes, as well as the role of the Mg^2+^-sensitive, TRPM6-, and TRPM7-like currents on the cardiac action potential.

## 2. Results

### 2.1. Expression of TRPM6 and TRPM7 in Cardiac Myocytes

**Figure 1:**
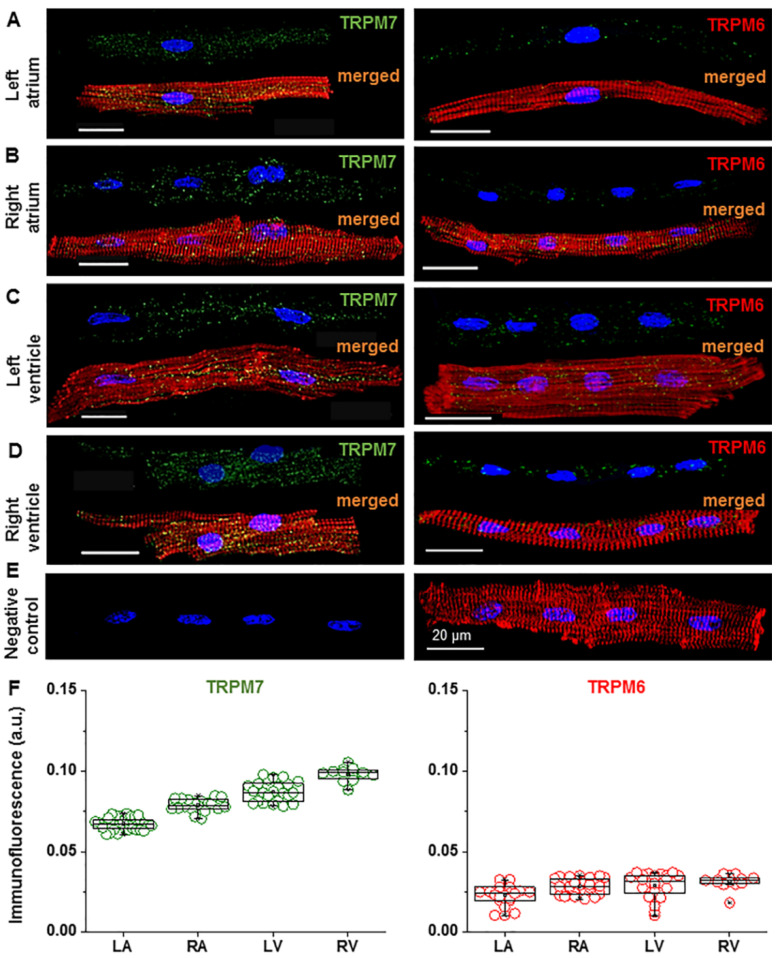
Immunofluorescence images suggesting the presence of TRPM6 and TRPM7 proteins in pig cardiomyocytes from different cardiac chamber walls. (**A**–**D**) Immunofluorescence of TRPM7 (left) and TRPM6 (right) in the left atrium (LA), right atrium (RA), left ventricle (LV), and right ventricle (RV) cardiomyocytes when using Alexa Fluor 488 for the TRPM7 and TRPM6 proteins (stained in green), Alexa Fluor 546 for the F-actin cytoskeleton (stained in red), and Hoechst 33342 for the nuclei (stained in blue). Scale bars indicate 20 µm. (**E**) Example of a negative control, where the primary antibody for TRPM6 and/or TRPM7 is not added, but the cardiomyocyte was subjected to Hoechst 33342 and Alexa Fluor 546. Under such conditions, only immunofluorescence of the nuclei (stained in blue) and F actin cytoskeleton (stained in red) is detected. Note: same cardiomyocyte in the left and right (merged image) panels (**F**) Quantification of the staining intensity of the immunodetected fluorescence of TRPM7 and TRPM6 in the four cardiac chamber walls: LA, RA, LV, and RV. The mean data is provided in arbitrary units (a.u.) (see Table 1). A blinded study design (with the origin or treatment of cells unknown to the investigator) was used for the detection of immunofluorescence during the various experimental conditions.

To show the presence of TRPM6 and TRPM7 proteins, we used the immunostaining of atrial and ventricular cardiomyocytes, performed after 2 h of cell isolation. Figure 1A–D shows confocal images of pig cardiomyocytes co-stained for the nucleus (blue), for either TRPM7 or TRPM6 protein (green), and for the F-actin-cytoskeleton (red), whereas Figure 1E shows a negative control (cardiomyocyte incubated in conditions similar to those of Figure 1A–D but with no primary antibody added in the incubation medium). All the cardiomyocytes displayed staining with antibodies for TRPM6 and TRPM7. Figure 1F shows the quantification of the immunodetected fluorescence of TRPM7 (left panel) and TRPM6 (right panel) in the four cardiac chamber walls: left atrium (LA), right atrium (RA), left ventricle (LV), and right ventricle (RV). Of note was the multinucleated nature of the cells, as previously noted for pig cardiomyocytes [24].

**Figure 2:**
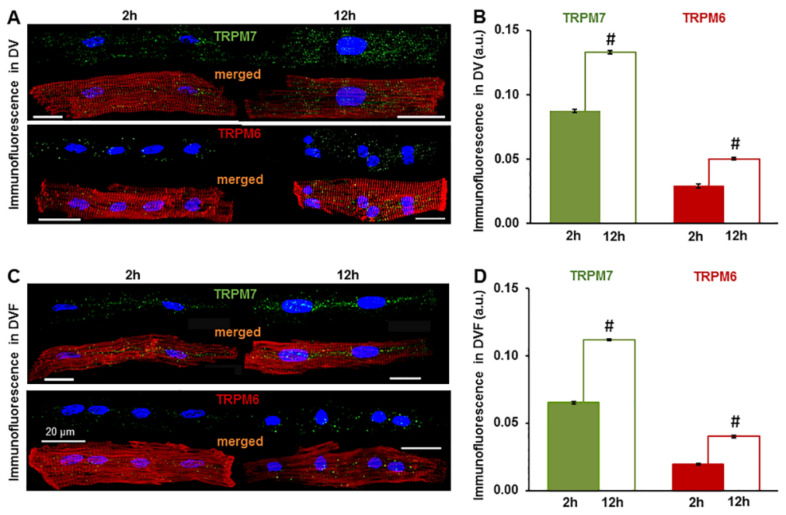
Comparison of the expression of TRPM6 and TRPM7 in left ventricular cardiomyocytes incubated for 2 h vs. 12 h in extracellular solutions with (**A**,**B**) and without (**C**,**D**) divalent cations (DV and DVF, respectively). (**A**,**C**) The cardiomyocytes were fixed after 2 h (filled columns) or 12 h (unfilled columns) of cell isolation: Alexa Fluor 488 for the TRPM7 and TRPM6 proteins (stained in green), Alexa Fluor 546 for the F-actin cytoskeleton (stained in red), and Hoechst 33342 for the nuclei (stained in blue). Scale bars indicate 20 µm. (**B**,**D**) Quantification of the intensity of the fluorescence expressed in arbitrary units (a.u.). # *p* < 0.001 expression after 12 h vs. 2 h of cardiomyocyte incubation.

We found that the measured level of expression of the TRPM6 and TRPM7 proteins was influenced by the cell incubation conditions, such as the presence and absence of extracellular divalent cations. The immunofluorescence level of both channel proteins in the cardiomyocytes from all the cardiac chamber walls was significantly higher following cell incubation in divalent cation-containing (DV) extracellular conditions vs. incubation in divalent cation-free (DVF) conditions (see Table 1). In addition, the expression was also increased when incubating cells for a longer period before cell fixation and exposure to the primary antibodies. Figure 2 shows that the mean fluorescence levels for the immunodetected TRPM6 and TRPM7 were significantly increased in LV cardiomyocytes kept for 12 h in solutions with (Figure 2A,B) or without (Figure 2C,D) divalent cations. TRPM7 increased from 0.087 ± 0.0013 a.u. to 0.133 ± 0.0011 a.u., *n* = 3–23, *p* < 0.001 in the DV solutions and from 0.065 ± 0.0009 a.u. to 0.112 ± 0.0006 a.u., *n* = 4–25, *p <* 0.001 in the DVF solutions. Under the same experimental conditions, the TRPM6 immunofluorescence increased from 0.029 ± 0.0015 a.u. to 0.050 ± 0.0012 a.u., *n* = 3–24, *p <* 0.001 and from 0.020 ± 0.0006 a.u. to 0.040 ± 0.0009 a.u., *n* = 7–21, *p <* 0.001 in the DV and DVF solutions, respectively. Qualitatively similar changes could be detected in the cardiomyocytes from the other cardiac chamber walls when incubated for 12 h (not illustrated).

### 2.2. Impact of Mg^2+^-Sensitive Currents on the Action Potential

To examine the role of Mg^2+^-sensitive currents on the electrical activity of cardiac myocytes, we recorded the total currents using a whole-cell voltage clamp, as well as the resting and action potentials using the current clamp. The cells were internally dialyzed and extracellularly perfused with solutions known to activate or inhibit the currents. The cells were internally dialyzed with either physiological levels of free intracellular [Mg^2+^] ([Mg^2+^]_i_ = 0.8 mM) or with the Mg^2+^-free solution ([Mg^2+^]_i_ ≈ 0 mM). The latter condition is known to cause a progressive activation of Mg^2+^-sensitive channels with time by removing the inhibition exerted by intracellular Mg^2+^ [15]. The cells were also extracellularly perfused with either physiological levels of [Mg^2+^] ([Mg^2+^]_o_ = 0.9 mM) or with high Mg^2+^ ([Mg^2+^]_o_ = 7.2 mM). High [Mg^2+^]_o_ is known to cause a complete suppression of any activated Mg^2+^-sensitive current [15].

**Figure 3:**
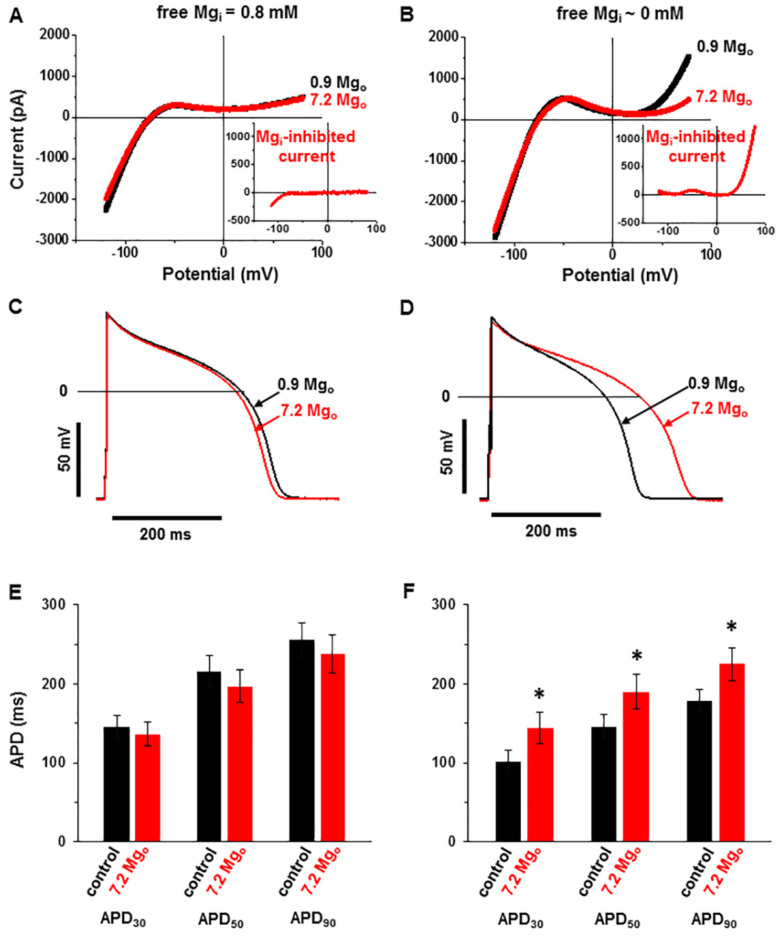
Effect of Mg^2+^-sensitive current activation on the action potential. (**A**,**B**) Whole-cell currents obtained by voltage ramps in cells dialyzed with 0.8-mM [Mg^2+^]_i_ (**A**) or 0-mM [Mg^2+^]_i_ (**B**) and superfused with extracellular solutions containing 0.9-mM [Mg^2+^]_o_ or 7.2-mM [Mg^2+^]_o_. Insets: the [Mg^2+^]_o_-sensitive currents calculated as the differences between currents in the presence of 0.9-mM [Mg^2+^]_o_ and those in 7.2-mM [Mg^2+^]_o_. Notice the outward-rectifying [Mg^2+^]_o_-sensitive current in the cell dialyzed with 0-mM [Mg^2+^]_i_. (**C**–**F**) The action potentials in the same cells as above, and the summary data of the action potential durations (APD) from all the cells dialyzed with 0.8-mM [Mg^2+^] (**C**,**E**) or 0-mM [Mg^2+^] (**D**,**F**) and the effect of raising the [Mg^2+^]_o_ from 0.9 mM (black) to 7.2 mM (red). The APD was measured at 30%, 50%, and 90% repolarization (APD_30_, APD_50_, and APD_90_, respectively). Notice the lengthening of the APD by high [Mg^2+^]_o_ in the cells dialyzed with low intracellular [Mg^2+^]. Pacing frequency: 1 Hz. * *p* < 0.05 for 7.2-mM vs. 0.9-mM [Mg^2+^]_o_. *n* = 9 for the cells dialyzed with 0.8-mM [Mg^2+^]_i_; *n* = 10 for the cells dialyzed with 0-mM [Mg^2+^]_i_.

Figure 3 shows the whole-cell currents and action potentials measured in the cells dialyzed with either 0.8-mM [Mg^2+^]_i_ (Figure 3A,C,E) or with 0-mM [Mg^2+^]_i_ (Figure 3B,D,F). In these two groups of cells, there was no difference in the resting membrane potentials, which were also not changed by raising [Mg^2+^]_o_ from 0.9 mM to 7.2 mM (for 0.8 mM [Mg^2+^]_i_, the resting membrane potential: −79.9 ± 2.6 mV in the control vs. −81.0 ± 2.1 mV in high [Mg^2+^]_o_; for 0 mM [Mg^2+^]_i_: −78.4 ± 2.8 mV in the control vs. −77.4 ± 3.7 mV in high [Mg^2+^]_o_, *p* = 0.308, ANOVA; *n* = 10).

Figure 3A,B illustrates the membrane currents recorded under a voltage clamp during a descending ramp, following a preceding depolarizing ramp meant to inactivate the voltage-dependent Na^+^- and Ca^2+^ currents (see Methods). The experiments were performed at 36 °C using K^+^-containing intra- and extracellular solutions. In cells dialyzed with physiological levels of [Mg^2+^]_i_ (0.8 mM; Figure 3A), the current–voltage relationship was characterized by a large inward current, consistent with the presence of the inward rectifier K^+^ current (I_K1_) at potentials negative to −75 mV, and the current–voltage relationship was relatively flat at more positive potentials. Raising [Mg^2+^]_o_ to 7.2 mM did not affect the current–voltage relationship, indicating that the high [Mg^2+^]_o_ did not affect the *I*_K1_ and that no other Mg^2+^-sensitive current was present (Figure 3A, inset). In the cells dialyzed with Mg^2+^-free solution (0 mM [Mg^2+^]_i_; Figure 3B), the current-voltage relationship was characterized by the presence of *I*_K1_ but also showed an outward rectifying current at positive potentials. The outward rectifying component was suppressed by raising [Mg^2+^]_o_ to 7.2 mM, indicating the presence of a Mg^2+^-sensitive current. This [Mg^2+^]_o_-sensitive current, calculated as the difference in the current–voltage relationships between the two conditions with different [Mg^2+^]_o_, displays outward-rectifying properties similar to those of the TRPM6 and TRPM7 currents (Figure 3B, inset).

After recording membrane currents under a voltage clamp, we switched to the current clamp mode to record the action potentials in the same cells. Pig cardiomyocytes offer an experimental advantage when studying factors that affect the action potential, since channels carrying the transient outward K^+^ current (I_to_) are not expressed in this species [25,26]. Under the control conditions, with 0.9 mM [Mg^2+^]_o_, the action potentials were generally shorter in cells dialyzed with Mg^2+^-free internal solution (compare Figure 3C,E vs. Figure 3D,F). In the cells dialyzed with physiological [Mg^2+^]_i_, raising [Mg^2+^]_o_ to 7.2 mM did not change the action potential durations during the stimulation at 1 Hz (APD; APD_30_: 145.5 ± 14.5 ms vs. 136.5 ± 15.1 ms, APD_50_: 216.6 ± 19.6 ms vs. 196.6 ± 21 ms, and APD_90_: 238 ± 48 ms vs. 231 ± 46 ms in high [Mg^2+^]_o_ vs. in the control; *p* > 0.05, paired *t*-test; *n* = 9; Figure 3E). In contrast, in cells dialyzed with Mg^2+^-free internal solution, raising [Mg^2+^]_o_ to 7.2 mM, caused a marked prolongation of the APD (APD_30_: from 100.4 ± 14.8 ms to 143.6 ± 19.9 ms, APD_50_: from 145.6 ± 16.1 ms to 189.8 ± 22.4 ms, and APD_90_: from 178.9 ± 14.2 ms to 225.2 ± 21.3 ms; *p* < 0.05, paired *t*-test; *n* = 10; Figure 3F). Taken together, these results show that the cardiac action potential is modulated by an outward-rectifying current activated by dialysis with zero [Mg^2+^]_i_ and suppressed by high [Mg^2+^]_o_. Furthermore, given that this current was absent in the cells dialyzed with 0.8 mM [Mg^2+^]_i_, it is likely due to TRPM7 or/and TRPM6.

### 2.3. Effects of High Extracellular [Mg^2+^] on I_Ca-L_ and I_K_

**Figure 4:**
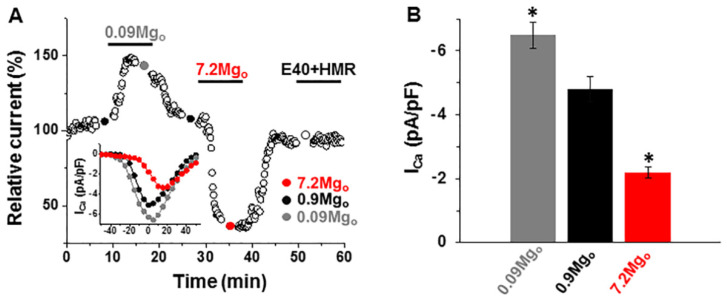
Effect of changing the external [Mg^2+^] on the L-type Ca^2+^ currents (I_Ca-L_). (**A**) Time diary of the amplitude of the L-type Ca^2+^ currents obtained by using depolarizing steps for various potentials in a cell dialyzed with 0.8-mM [Mg^2+^] and superfused with extracellular solutions containing 0.09-mM, 0.9-mM, or 7.2-mM [Mg^2+^]. Notice the increase vs. suppression of the I_Ca-L_ amplitude by low vs. high [Mg^2+^]_o_ and the lack of effect of the I_K_ inhibitors (E4031/HMR1556). Bottom inset: The current–voltage relations obtained by the depolarizing steps to the potentials ranging from −50 mV to +50 mV in the same cell. (**B**) Summary data of the peak I_Ca-L_ amplitude and the effects of lowering and raising the [Mg^2+^]_o_. * *p* < 0.05 vs. 0.9-mM [Mg^2+^]_o_ (*n* = 8–13).

Considering that the changes in the action potential produced by high [Mg^2+^]_o_ mainly affected the plateau and repolarization phases, we also examined the effect of [Mg^2+^]_o_ on other currents that play a role during these phases, such as the L-type Ca^2+^ current (I_Ca-L_) and the delayed rectifier K^+^ current (I_K_). The analysis was done in cells dialyzed with 0.8-mM [Mg^2+^]_i_ to inhibit the Mg^2+^-sensitive currents. For the measurements of I_K_, K^+^-containing intracellular and extracellular solutions were used, and nifedipine (25 µM) was included in the external solution, whereas, for the measurements of I_Ca-L_, K^+^ was replaced by Cs^+^ in the external and pipette solutions, and 10-mM BAPTA was used in the pipette solution instead of EGTA. Figure 4A shows the bidirectional effect of the [Mg^2+^]_o_ alteration on I_Ca-L_. Where lowering the [Mg^2+^]_o_ by 10-fold to 0.09 mM reversibly increased the amplitude of I_Ca-L_ continuously monitored at 0 mV (I_Ca-L_ measured at the peak level: from −5.4 ± 0.43 pA/pF to −6.5 ± 0.41 pA/pF; *p* < 0.05, paired *t*-test; *n* = 8; Figure 4B), raising the [Mg^2+^]_o_ to 7.2 mM reversibly decreased the amplitude of I_Ca-L_ (peak I_Ca-L_: from −4.8 ± 0. 39pA/pF to −2.2 ± 0.17 pA/pF; *p* < 0.001, paired *t*-test; *n* = 13; Figure 4B; see reference [27]). As expected, the I_K_ blockers E4031 and HMR1556 had no effect on the I_Ca-L_ (Figure 4A). When wanting to test the consequence of an eventual modulation of the I_Ca-L_ by extracellular Mg^2+^, we kept E4031 constantly from the beginning of the experiment. The I_Ca-L_–voltage relationships (see Figure 4A, inset) indicate that the [Mg^2+^]_o_ changes shifted the activation curve of I_Ca-L_ (to more negative potentials upon lowering the [Mg^2+^]_o_ and to more positive potentials upon raising [Mg^2+^]_o_; see reference [27]).

**Figure 5:**
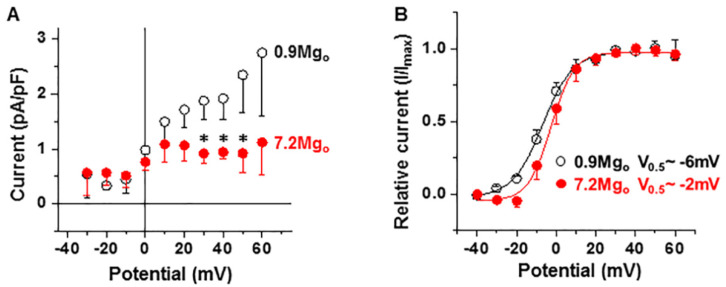
Effect of an increase of extracellular [Mg^2+^] on the total I_K_. (**A**) The current–voltage relationship of the fully activated I_K_ and the effect of raising the [Mg^2+^]_o_. Notice the suppression of I_K_ by high [Mg^2+^]_o_. * *p* < 0.05 (*n* = 4). (**B**) Activation curves of I_K_ calculated from the tail currents (*n* = 4).

Figure 5 shows that raising [Mg^2+^]_o_ also decreased the magnitude of the total I_K_ (Figure 5A at +30 mV to +50 mV *p* < 0.05 for 7.2-mM [Mg^2+^]_o_ vs. the control, paired *t*-test; *n* = 4) and only mildly shifted the activation curve to the right (voltage at the half-maximal current V_0.5_: from ≈−6 mV to ≈−2 mV; *n* = 4; Figure 5B).

**Figure 6:**
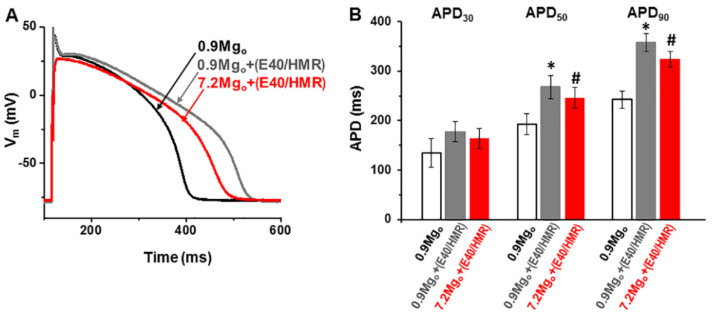
Effect of a partial I_K_ block and high [Mg^2+^]_o_ on the action potential. (**A**) APs recorded at 36 °C using K^+^-containing intra- and extracellular solutions under perforated patch conditions and initially superfused with control extracellular solutions containing 0.9-mM [Mg^2+^]. A combination of HMR1556 (100 nM) and E4031 (500 nM) was then added in 0.9-mM [Mg^2+^]_o_ before applying 7.2-mM [Mg^2+^]_o_ together with the drugs. (**B**) Summary data of the AP duration (APD) at 30%, 50%, and 90% repolarization (APD_30_, APD_50_, and APD_90_, respectively) measured under the control conditions (unshaded column) in the presence of the I_K_ inhibitors (grey column) and in the presence of the I_K_ inhibitors but with the [Mg^2+^]_o_ raised to 7.2 mM (red column). * *p* < 0.05 for the presence vs. absence of the I_K_ inhibitors (in 0.9-mM [Mg^2+^]_o_). # *p* < 0.05 for 7.2-mM vs 0.9-mM [Mg^2+^]_o_ in the presence of the I_K_ inhibitors (*n* = 4). Notice the shortening of the APD_50_ and APD_90_ by high [Mg^2+^]_o_ in the presence of the I_K_ blockers.

Since high [Mg^2+^]_o_ did not change the APD in cells dialyzed with 0.8-mM [Mg^2+^]_i_ (see Figure 3C,E), it is possible that there are two opposing and counterbalancing effects of high [Mg^2+^]_o_ on the I_Ca-L_ and I_K_. To test for this possibility, we applied high [Mg^2+^]_o_ on cells in which the I_K_ was partly blocked to offset such a balance. We initially performed preliminary tests to determine the optimum concentrations of the I_K_ inhibitors (i.e., HMR1556 for the slow I_K_ component *I*_Ks_ and E 4031 for the rapid component *I*_Kr_) effective in producing a partial block of I_K_ without excessively prolonging the APD (not illustrated). Figure 6A shows the typical changes in action potentials in a perforated cell, whereas the summary data from four cell are presented in Figure 6B. Under the control conditions with 0.9-mM [Mg^2+^]_o_, the addition of small concentrations of I_K_ inhibitors (100-nM HMR1556 and 500-nM E4031) lengthened the APD, as expected from a decrease of the repolarizing K^+^ current (at a pacing rate of 1 Hz, APD_30_: from 134.6 ± 28.5 ms to 177.8 ± 19.9 ms, APD_50_: from 192.9 ± 20.9 ms to 267.4 ± 24.1 ms, and APD_90_: from 242.4 ± 17.5 ms to 358.0 ± 18.3 ms; * *p* < 0.05, except for the APD_30_, for which *p* > 0.05, paired *t*-test; *n* = 4). Consistent with a decreased relative contribution of I_K_, raising the [Mg^2+^]_o_ to 7.2 mM in the presence of the I_K_ blockers decreased the APD (to 163.9 ± 20.9 ms, to 246.0 ± 21.1 ms, and to 323.5.0 ± 15.5 ms for APD_30_, APD_50_, and APD_90_, respectively), indicating a predominant effect of I_Ca-L_ suppression by high [Mg^2+^]_o_ under these conditions. Qualitatively similar results were obtained in three other cells dialyzed with 0.8-mM [Mg^2+^]_i_ under the ruptured cell membrane conditions (not illustrated). These results suggest that the lack of effect of raising [Mg^2+^]_o_ on the APD in cells dialyzed with 0.8-mM [Mg^2+^]_i_ reflects a balance in the effects of the decreases of both I_Ca-L_ and I_K_ on repolarization.

## 3. Discussion

The results from the present study suggest the presence of TRPM6 and TRPM7 proteins in cardiac myocytes, given that immunofluorescent activity was detected in cells treated with the anti-TRPM6 or anti-TRPM7 antibody, but in contrast, no such activity was detected in the negative controls. Furthermore, the results showed that the cardiac myocyte action potential duration (APD) was shortened in the presence of a current likely due TRPM6 or TRPM7 in that it was activated by dialysis with low [Mg^2+^]_i_, was absent in cells dialyzed with 0.8-mM [Mg^2+^]_i_ and was suppressed by high [Mg^2+^]_o_. Thus, the results are consistent with our previous proposal that the Mg^2+^-sensitive channels in the heart are likely due to TRPM6 and/or TRPM7 proteins [15] and with a possible role of the cardiac Mg^2+^-sensitive channels in modulating the electrical activity.

### 3.1. Molecular Candidate for Mg^2+^-Sensitive Channels

Many TRP proteins form nonselective cation channels and are, therefore, the molecular candidates for similar native channels [28]. In determining the involvement of TRPs expressed in the heart, the inhibition by Mg^2+^_i_ is a key distinguishing feature. For the TRPC4/C5 channels, Mg^2+^_i_ causes a voltage-dependent partial block of the outward currents and results in a doubly rectifying current–voltage relationship [29,30]. Such a Mg^2+^_i_ effect on the TRPC4/C5 channels is different from the voltage-independent slow inhibition seen in the Mg^2+^-sensitive channels [15,31]. Rather, the Mg^2+^_i_ inhibition and the biophysical characteristics of the cardiac channels [15] resemble those of the TRPM6 and TRPM7 channels [16,19]. Our detection of TRPM6 and TRPM7 proteins by immunofluorescence suggests that the channels are expressed in both the atrial and ventricular chambers. It appears that the TRPM6 and TRPM7 channel proteins were present in both the surface membrane and intracellularly. However, where exactly the proteins are located intracellularly could not be determined in the present study. Nonetheless, a novelty of the present findings is that TRPM6 was immunodetected in pig myocytes (but see Limitations of our Study below), whereas TRPM6 expression has been reported to be lacking in other studies of cardiac tissues [32]. As such, the present results suggest that TRPM6 and TRPM7 proteins are the most likely constituents of the Mg^2+^-sensitive cardiac channels.

There are, however, slight differences in the divalent cation permeability profiles between the cardiac Mg^2+^-sensitive channels [15] and overexpressed TRPM7 channels [16]. It is possible that, even though the core structure of the channel may be the same, different channel subunits or regulatory units may occur in the native cells. Furthermore, the TRPM7 channel activity has been shown to be altered by heteromultimeric interactions with TRPM6 [33]. Nevertheless, in order to ascertain the cardiac channel identity, further studies are still required to correlate the activity of the TRPM6 and TRPM7 proteins detected in myocytes with the Mg^2+^-sensitive current.

### 3.2. Modulation of Cardiac Electrical Activity

The possible contribution of the Mg^2+^-sensitive current to cardiac electrical activity is unknown. Like in other cells, nonselective cation channels contribute to the resting membrane potential in the heart [34]. These may include Mg^2+^-sensitive channels. Given the small Mg^2+^-sensitive inward current and the large *I*_K1_ at a negative potential in the heart, the Mg^2+^-sensitive channels would not be expected to contribute significantly to the background non-K^+^ permeability in resting cardiomyocytes. This is consistent with our findings here, showing no effect of raising the extracellular Mg^2+^ concentration on the resting membrane potential. However, in other tissues such as vascular smooth muscle, the contribution of nonselective cation channels, some of which resemble TRPM6 and TRPM7, could be more important [35,36].

Cation nonselective channels may also contribute to shaping the cardiac action potential. In atrial cells, a stretch-activated cation nonselective channel has been shown to contribute to both the resting and action potentials [37]. Since the net ion current is small during the plateau phase of the action potential, changes in the large monovalent cation effluxes through Mg^2+^-sensitive channels at positive potentials may have significant effects on the action potential. Consistent with such an expectation, our present results showed that the activation of the cardiac Mg^2+^-sensitive channels shortened the APD. It is unlikely that the APD shortening is due to a larger outward *I*_K1_ following the removal of the Mg^2+^_i_ block in low [Mg^2+^]_i_ conditions, since endogenous polyamines would still continue to provide a sufficient block of the outward *I*_K1_ [38] and since high Mg^2+^_o_ suppressed the outward current while having no effect on the inward currents. Our results suggest that there is practically no contribution of TRPM6 and TRPM7 currents to the AP when [Mg^2+^]_i_ is at the physiological levels. This result is consistent with a lack of changed currents through mutant TRPM7 channels expressed in cultured cardiomyocytes on the action potentials measured in such cells [39].

### 3.3. Clinical Implications

A key question concerns the conditions under which the Mg^2+^-sensitive channels may contribute to cardiac electrical activity. Under the physiological conditions (free [Mg^2+^]_i_ 0.8 mM; IC_50_ ≈ 0.25 mM; [15,31]), the channels are expected to be substantially inhibited by Mg^2+^_i_. However, the Mg^2+^-sensitive current may contribute to APD if the sensitivity to [Mg^2+^]_i_ is decreased by regulatory processes or channel mutations or if [Mg^2+^]_i_ is decreased to low levels by disease conditions such as chronic hypomagnesaemia. Indeed, the TRPM7 and related native channels show constitutive activity, some of it in cells where the intracellular ion composition (including [Mg^2+^]_i_) remains relatively unperturbed [16,18,40], suggesting the presence of other regulatory processes. In cardiac cells, besides [Mg^2+^]_i_, the Mg^2+^-sensitive channels are also regulated by factors such as the pH change and membrane phospholipid metabolism [14,15,41], but it is still not known whether such regulatory processes can induce the constitutive activity of the channels. In the present study, we show that the expression of the TRPM6 and TRPM7 channels is modulated by incubation in divalent cation-free extracellular conditions. Thus, the contributions of the Mg^2+^-sensitive current to the AP may vary depending on the status of the extracellular divalent cation homeostasis.

Earlier studies demonstrated TRPM7 activation by free oxygen radicals during prolonged neuronal ischemia [42]. To date, the functional role of TRPM7 and especially that of TRPM6 are less clearly understood in heart cardiomyocytes compared to vascular smooth muscle cells [43], neurons, or other cell types [44,45]. The molecular and electrophysiological characterizations of TRPM7 in the heart have focused predominantly on cardiac fibroblasts. TRPM7 activation in human atrial fibroblasts leads to fibrogenesis and atrial fibrillation [46]. Additionally, the variability of the TRPM7 current density in human right atrial cardiomyocytes is related to the clinical history, being higher in disease conditions such as atrial fibrillation [47,48] and in ischemic heart disease [48]. Very recently, we also demonstrated the presence and co-expression of both TRPM6 and TRPM7 in cardiomyocytes from the four chamber walls of the human heart [22] and also showed that ischemic heart disease may increase their expression, suggesting that chanzymes are involved in the pathophysiology of the disease.

### 3.4. Limitations of the Study

Given the current uncertainty about the detection of TRPM6 in cardiac cells, it is important to examine how convincing the evidence is for the presence of TRPM6 in our study.

The present study relied on immunostaining to detect the presence of TRPM7 and TRPM6. Previous studies have demonstrated limitations of antibody staining methods to determine the expression of TRP channels (e.g., see [49]). The specificity of the commercial antibodies used in our study was not directly tested, e.g., using blocking peptides or using cells lines in which the TRPM7 and TRPM6 genes were knocked out, silenced or overexpressed.

Our results, using swine cardiomyocytes, are in concordance to human TRPM6 and TRPM7 immunodetected fluorescence distribution, which also suggested both ion channel expression in the walls of all cardiac chambers [22]. In that study RT-PCR confirmed the presence of mRNA for both TRPM7 and TRPM6, hence supporting the immunostaining data, but the interpretation of RT-PCR also has limitations due to possible contamination by non-cardiomyocyte cells. We used on pig myocytes the same antibodies as in that study. Although these antibodies are recognized to work on human proteins, a key limitation of the present study is that there is no previous evidence of their specificity in pig cells and there are no readily available pig-specific TRPM6 antibodies in the market. Given that there is a great amount of antibody recognition between human- and pig epitopes in cardiac tissue [50], we used the same TRPM6 antibody (designed for human, mouse, and rat), which being polyclonal, would also be able to bind to epitopes in other species. Although TRPM6 was detected in pig cardiomyocytes but not in negative controls, the specificity of the antibody in pig still requires verification in future studies.

An important limitation of the electrophysiological study is that there are, as yet, no known specific pharmacological blockers available for Mg^2+^-sensitive- and related channels. Presently, we used the block by Mg^2+^_o_ as an extracellular tool to isolate the Mg^2+^-sensitive current. There was therefore a possible confounding issue in that Mg^2+^_o_ could also suppress I_Ca-L_ and I_K_. In other studies, the Mg^2+^_o_ effect on I_K_ was more prominent on tail currents during repolarization [51], and increasing Mg^2+^_o_ caused either a lengthening or a shortening of APD, depending on the level of Mg^2+^_o_ [52]. However, in the present study, the Mg^2+^_o_ effects on action potentials that were due to I_Ca-L_ and I_K_ seemed to balance each other out sufficiently for their effects to be isolated from those on the Mg^2+^-sensitive current.

### 3.5. Conclusion

In conclusion, our results show the presence of TRPM7 and, to a certain extent, suggest that of TRPM6 proteins in pig cardiomyocytes, making these proteins the molecular candidates for cardiac Mg^2+^-sensitive channels. The activation of the Mg^2+^-sensitive channels shortened the cardiomyocyte APD. Although the pathophysiological conditions in which the channels are activated remain unclear, the effect of the Mg^2+^-sensitive current on the APD may be important in understanding the therapeutic processes in which Mg^2+^ is empirically used to treat arrhythmias, as well as in linking the changes in Mg^2+^ homeostasis to other cardiac disease conditions.

## 4. Materials and Methods

We used isolated, single cardiomyocytes of a pig heart. This study was carried out in compliance with the Guide for the Care and Use of Laboratory Animals (NIH). All experiments were performed according to the European Community guiding principles and approved by the State Food and Veterinary Service of the Republic of Lithuania (No. G2-68, 21 June 2017) and by the Belgian laboratory license No. LA-1210253. 

### 4.1. Cell Isolation

The methods used for the dissociation of pig cells have been described before [15,25]. In short, for electrophysiology studies, a piece of the left ventricular wall was excised, and its supplying artery was cannulated and perfused at 37 °C and at constant pressure for 30 min with an oxygenated Ca^2+^-free Tyrode solution, followed by a 20- to 25-min perfusion with a Ca^2+^-free Tyrode solution containing 0.1-mg mL^−1^ protease (type XIV, Sigma-Aldrich, St. Louis, MO, USA) and 1.4-mg mL^−1^ collagenase (type A, Boehringer-Mannheim, Mannheim, Germany). After a 15-min washing perfusion with a 0.18-mM Ca^2+^ Tyrode solution, the tissue was removed from the perfusion and was cut into small pieces. The cells were dispersed by gentle mechanical agitation. [Ca^2+^]_o_ was raised in a stepwise manner, and the cells were stored at room temperature (21–22 °C). Ca^2+^-tolerant rod-shaped ventricular myocytes with clear striations were selected for the electrophysiological studies.

For the immunofluorescence studies, the cardiomyocytes were isolated from a small tissue specimen as previously described [53]. In short, each tissue specimen (LA, RA, LV, and RV) was fine-cut in an oxygenated nominally Ca^2+^-free Tyrode solution (see the composition below) supplemented with 3-mg/mL 2,3-butanedione monoxime, which was washed out 2 to 3 times before the enzyme application. The cardiac tissue chunks were transferred to a beaker with nominally Ca^2+^-free Tyrode solution supplemented with 1-mg/mL bovine serum albumin (BSA), 1-mg/mL collagenase (215 U/mg, type 2; Worthington Biochemical Corporation, Lakewood, NJ, USA), and 0.5-mg/mL protease (7–14 U/mg, type XXIV; Sigma-Aldrich, St. Louis, MO, USA) and continuously bubbled with 100% O_2_. After 30 min of shaking in a water bath at 37 °C, the solution with both enzymes was replaced by fresh solution containing only collagenase (1 mg/mL) and shaken until cardiomyocytes appeared in the aliquots obtained from the mixture. When the yield appeared to be optimal, the leftovers of the tissue chunks were resuspended in nominally Ca^2+^-free Tyrode and gently subjected to trituration by suction with a pipette. The cell suspension was filtered, centrifuged, and washed 2 to 3 times either with normal (divalent cation-containing) or with divalent cation-free Tyrode solution and stored at room temperature.

### 4.2. Electrophysiology

Whole-cell currents were recorded under a voltage clamp, and the action potentials were recorded under a current clamp at 36 °C within 4 h after cell isolation. The membrane currents of interest were measured using 2-s descending voltage ramps from +80 mV to −120 mV applied every 10 s after a 600-ms pre-step at 0 mV from a holding potential of −80 mV, designed to inactivate the voltage-dependent Na^+^ and L-type Ca^2+^ currents. For measuring the L-type Ca^2+^ currents (I_Ca-L_), 400-ms depolarizations to various potentials were given after a 400-ms pre-step at −40 mV, designed to inactivate the voltage-dependent Na^+^ current. The total delayed rectifier K^+^ currents I_K_ were measured using voltage steps from a holding potential of −40 mV to various positive potentials, and the tail currents were then measured upon reverting to −50 mV. The action potentials were measured by stimulating with a 2-ms rectangular pulse at a frequency of 1 Hz. The action potential duration (APD) was measured at 30% (APD_30_), 50% (APD_50_), and 90% (APD_90_) repolarization. The current and voltage protocols were generated and data recorded online using the Axopatch 200B amplifier and pClamp 8.1 software via the Digidata 1322A acquisition system (Axon instruments, Union City, CA, USA).

### 4.3. Immunofluorescence

Enzymatically dissociated cardiomyocytes were allowed to settle on the bottom of 8-chamber slides. The cells were permeabilized and incubated with primary rabbit polyclonal anti-TRPM7 (#ACC-047; Alomone Labs, Jerusalem, Israel) or rabbit polyclonal anti-TRPM6 antibody (#ACC-046; Alomone Labs, Jerusalem, Israel) diluted (1:200) in PBS containing 3% BSA in blocking buffer overnight at 4 °C. The TRPM6 and TRPM7 antibodies were obtained from the same company in order to minimize the possibility of cross-reactivity during immunolabeling. For the negative controls, incubation with the primary antibody was omitted to check for nonspecific binding of the secondary antibody. The cells were incubated for 1 h with a fluorescently labeled secondary antibody (donkey anti-rabbit IgG; Alexa Fluor^®^ 488 conjugate; A21206, Invitrogen, Thermo Fisher Scientific, Rockford, IL, USA; dilution 1:200) co-stained (for 20 min) with Phalloidin-Alexa Fluor^®^ 546 (A22283, Invitrogen, Thermo Fisher Scientific, Waltham, MA, USA; dilution 1:100) and with Hoechst 33342 (B2261, Sigma-Aldrich, St. Louis, MO, USA; for 10 min) for labeling of the F-actin cytoskeleton and of the nucleus, respectively. Cardiomyocytes were visualized under a confocal laser scanning microscope (Olympus BX61, Olympus Corporation, Tokyo, Japan) from which images were taken using the same scanning parameters for both the TRPM7 and TRPM6 proteins in all the cardiomyocyte preparations.

### 4.4. Solutions and Drugs

The standard Tyrode solution used during cell dissociation contained (in mM): 135 NaCl, 5.4 KCl, 0.9 MgCl_2_, 1.8 CaCl_2_, 0.33 NaH_2_PO_4_, 10 HEPES, and 10 glucose; the pH was adjusted to 7.4 with NaOH. During the patch clamp measurements, the cells were superfused with a solution of similar composition, except that, when necessary, K^+^ was replaced by Cs^+^, and the Mg^2+^ levels were changed. The standard pipette solution was contained (in mM): 155 KCl, 5.5 MgCl_2_, 5 Na_2_ATP, 1 EGTA, 0.1 Na_2_GTP, and 5 HEPES (pH 7.25; adjusted with KOH) and was modified by changing the levels of Mg^2+^, by replacing K^+^ with Cs^+^, or by substituting EGTA with BAPTA. The phosphate-buffered saline (PBS) used in immunofluorescence contained (in mM): 155.2 NaCl, 2.71 Na_2_HPO_4_·2H_2_O, and 1.54 KH_2_PO_4_ (pH 7.4; adjusted with NaOH).

HMR1556 was from Aventis Pharma, Frankfurt Am Main, Germany, and E-4031 was from Tocris, Bristol, UK. All the other drugs or chemicals were from Sigma-Aldrich (Bornem, Belgium) or Merck (Darmstadt, Germany). Nifedipine was prepared as a stock solution in ethanol and was protected from light, whereas HMR1556 was prepared in dimethyl sulfoxide (DMSO). All the other chemicals were dissolved in water.

### 4.5. Data and Statistical Analyses

An electrophysiology data analysis was performed using Clampfit 8.2 (Axon Instruments, Union City, CA, USA) and Origin 7 (Microcal, Northampton, MA, USA).

The distribution of the immunofluorescence was analyzed using the Olympus Fluoview FV1000 (Olympus Corporation, Tokyo, Japan) and ImageJ software. Sixty-times magnifying (60×) of the oil immersion objectives were utilized for all the acquisitions. The images were presented as stacks of 8–10 slices at a fixed intensity. The cardiomyocyte area (pixels) and fluorescence intensity were measured in stacks using Imaris software (Bitplane AG, Zurich, Switzerland). The immunodetected TRPM6 and TRPM7 protein levels were calculated by the formula: fluorescence intensity × 1000/cell area. In order to reduce the effect on any statistical confounder, the same parameters for fluorescence intensity detection (image acquisition control and spectral settings, etc.) were always applied. In addition, the immunofluorescence reading was blinded, as the conditions used to keep the cells were unknown to the person performing the reading.

The average data were presented as the mean ± standard error of the mean (S.E.M) or box plots, with *n* indicating the number of cells studied under each condition. The means were compared using the two-tailed Student’s *t*-test, whereas differences among the multiple groups were evaluated using an analysis of variance (ANOVA). *p* ≤ 0.05 was taken as the threshold for statistical significance.

## Figures and Tables

**Table 1:** Immunofluorescence signals of the TRPM7 and TRPM6 proteins in pig hearts.

Heart Chamber	TRPM7 Signal (a.u.)		TRPM6 Signal (a.u.)	
	DV	DVF	DV	DVF
LA	0.067 ± 0.0006 *n* = 37	0.053 ± 0.0009 # *n* = 13	0.023 ± 0.0013 *n* = 18	0.016 ± 0.0014 # *n* = 20
RA	0.079 ± 0.0009 *n* = 21	0.059 ± 0.0010 # *n* = 5	0.029 ± 0.0008 *n* = 32	0.018 ± 0.0011 # *n* = 4
LV	0.087 ± 0.0013 *n* = 23	0.065 ± 0.0009 # *n* = 25	0.029 ± 0.0015 *n* = 24	0.020 ± 0.0006 # *n* = 21
RV	0.098 ± 0.0013 *n* = 12	0.076 ± 0.0016 # *n* = 5	0.032 ± 0.0012 *n* = 14	0.025 ± 0.0020 # *n* = 3

TRPM7 and TRPM6—transient receptor potential melastatin type 7 and 6 channels, LA—left atrium, RA—right atrium, LV—left ventricle, RV—right ventricle, DV—extracellular divalent cations, DVF—extracellular divalent cation-free, *n*—number of cells, a.u.—arbitrary unit, and #—*p* < 0.001 for DV vs. DVF.
